# Epidermotropic Metastatic Prostate Adenocarcinoma With Florid Squamous Transformation/Differentiation and Associated Acantholysis. An Unusual Clinicopathologic Entity

**DOI:** 10.1155/crdm/5879396

**Published:** 2026-09-03

**Authors:** Sara J. Hoffman, Kaviyon Sadrolashrafi, Narciss Mobini

**Affiliations:** ^1^ Department of Pathology and Laboratory Medicine, UC Davis Health, Sacramento, California, USA, lhsc.on.ca; ^2^ Department of Medicine, Division of Dermatology, David Geffen School of Medicine at UCLA, Los Angeles, California, USA, ucla.edu; ^3^ Department of Internal Medicine, Kirk Kerkorian School of Medicine at UNLV, Las Vegas, Nevada, USA; ^4^ Associated Pathologists Chartered, Quest Diagnostics, Las Vegas, Nevada, USA

## Abstract

Prostate adenocarcinoma is the most common carcinoma in men. Cutaneous metastasis of prostate adenocarcinoma, however, is rare, accounting for less than 1% of metastatic cutaneous carcinomas. This cutaneous spread most often occurs in the inguinal area and the penis, followed by the abdomen, head and neck, chest, extremities, and back. Metastatic prostate adenocarcinoma generally has a poor prognosis, emphasizing the importance of timely identification. We report a patient with an unusual clinicopathologic presentation of metastatic prostate adenocarcinoma manifesting as a scaly plaque on the left shin. This lesion was histopathologically characterized by marked epidermotropism, squamous differentiation, and acantholytic features, with a negative reaction to prostate‐specific antigen and prostatic acid phosphatase immunostains. When evaluating metastatic cutaneous carcinomas displaying epidermotropism and squamoid appearances or squamous differentiation, it is essential to maintain a high index of suspicion and inquire about a history of prostate carcinoma. This history is especially relevant if hormonal therapy was utilized as part of the patient’s management.

## 1. Introduction

Prostate carcinoma is the most common type of cancer in men and ranks second only to lung cancer as the leading cause of cancer‐related deaths among males in the United States [1]. 95% of all prostate carcinomas are adenocarcinomas [2]. According to the CDC, cases from 2016 to 2020 show that 70% of prostate cancers were diagnosed at the localized stage (i.e., not spreading beyond the prostate), while 13% were classified as regional stage cancers, meaning they had spread to nearby lymph nodes or tissues organs. Eight percent were found at a distant stage (i.e., spread to distant parts of the body), while 9% were unknown [3]. Cutaneous metastasis of prostate cancer is very rare, and fewer than 80 cases have been reported in the literature [4]. In a retrospective study of 7316 cancer patients, 5% showed skin involvement. More recent studies suggest the overall rate to be about 1%–4%; of those, 0.3% is due to prostate adenocarcinomas [5, 6]. The diagnosis of metastatic prostate adenocarcinoma requires a high index of suspicion and is made through a strong clinicopathologic correlation. The role of immunohistochemistry (IHC) is extremely useful to help determine the origin of the metastatic disease [7]. Prostate‐specific antigen (PSA) is the most common immunohistochemical stain used to diagnose prostate adenocarcinoma [8]. Although a highly sensitive marker, false negative cases have been reported, particularly in poorly differentiated tumors or hormone‐treated cases of prostate carcinoma. In situations when PSA is negative, the prostatic acid phosphatase (PAP or PSAP) immunostain may be utilized to establish the diagnosis [9, 10]. Although serum PSA levels can be elevated, cutaneous deposits may fail to express PSA and show a positive reaction to PAP [7]. On rare occasions, including cases of treated prostate cancer, there may be an associated loss of reactivity for both PSA and PAP. In these situations, more recently available markers, such as prostate‐specific membrane antigen (PSMA) and NKX3.1, may provide benefit [11–14]. Squamous differentiation is a rare occurrence in prostate carcinoma. Squamous transformation after initial treatment of a primary prostate adenocarcinoma is even less common [15]. We report an unusual presentation of cutaneous metastatic prostate adenocarcinoma with negative PSA and PAP and positive PSAP and NKX3.1 reactions, in a highly squamoid appearance and acantholysis.

## 2. Case Report

**FIGURE 1:**
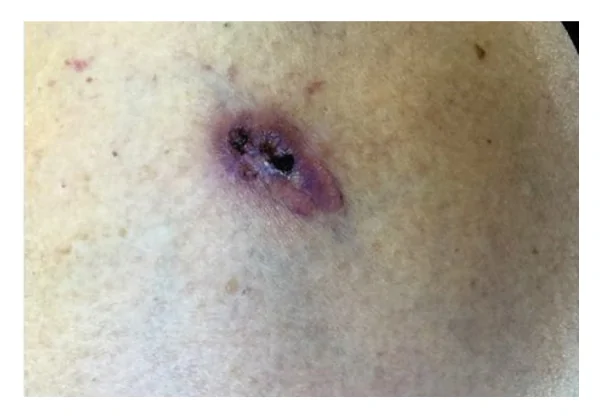
Erythematous and scaly plaque on the left shin.

**FIGURE 2:**
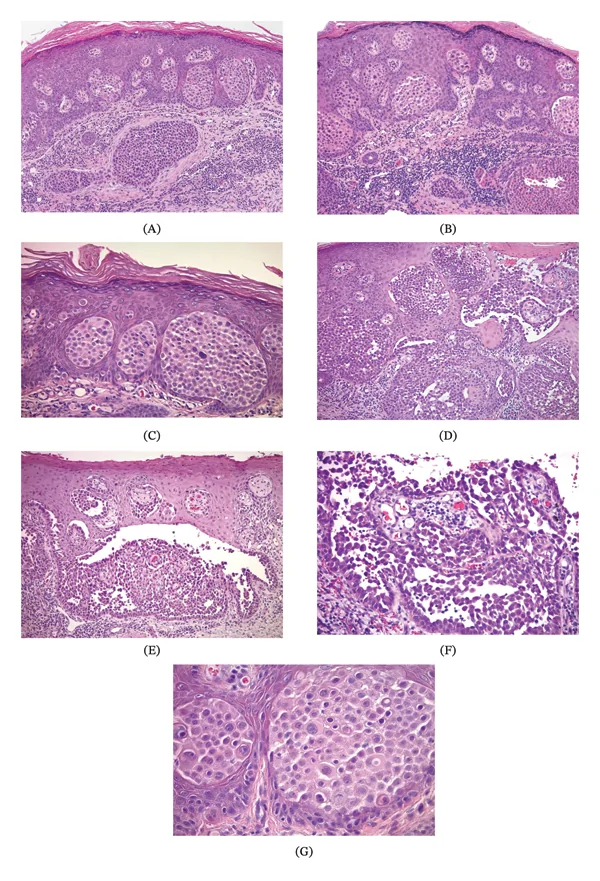
(A, B) Poorly differentiated metastatic carcinoma, both in the dermis and epidermis. 100x, H&E. (C) Metastatic carcinoma. Epidermotropic component. 200x, H&E. (D, E) Metastatic carcinoma. Epidermotropic component with acantholysis. 100x, H&E. (F) Metastatic carcinoma. Marked acantholysis. 200x, H&E. (G) Squamous differentiation of the cells with intercellular bridges. 400x, H&E.

**FIGURE 3:**
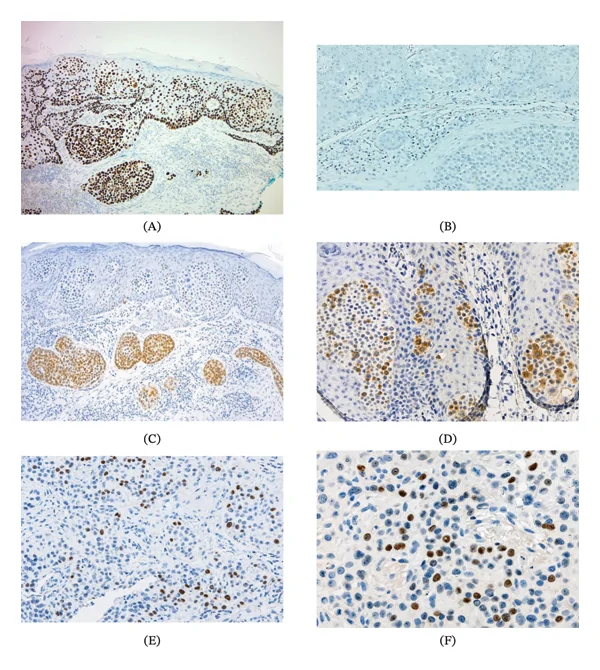
(A) P63 nuclear positivity of the neoplastic cells in the dermis and epidermis. 100x. (B) Negative reaction for PSA in the neoplastic cells. 100x. (C, D) PSMA cytoplasmic and membranous positivity 100x and 400x, respectively. (E, F) NKX3.1 nuclear positivity 100x and 400x, respectively.

A 70‐year‐old male presented to his primary care physician with a new‐onset keratotic plaque on his left shin (Figure 1). A biopsy was performed, with the clinical impression of “possible solar keratosis or basal cell carcinoma”. Histopathologic evaluation revealed a malignant neoplasm in the dermis and the epidermis with prominent pagetoid/epidermotropic morphology, characterized by discrete nests and aggregates of mostly cohesive epithelial cells displaying enlarged round to oval vesicular nuclei and prominent nucleoli and moderate‐to‐ample amounts of pale to bright eosinophilic cytoplasm associated with many typical and atypical mitotic figures (Figures 2A,B). Although most of the basal layer was intact, it was focally involved. In many foci, there was evidence of acantholysis with dissociation of cellular aggregates (Figures 2C–F). Squamous differentiation was evident in many foci with easily identifiable intercellular bridges (Figure 2G). Because of these unusual histopathologic findings, a panel of immunohistochemical stains with their appropriate controls was performed that revealed the constituent malignant cells in the dermis and epidermis to be positive for cytokeratin AE1/AE3 (not shown), p63 (Figure 3A), and p40. There was a negative reaction for cytokeratin 7, cytokeratin 20, CK 5/6, S100, and carcinoembryonic antigen (CEA). Due to atypical features uncharacteristic of primary squamous cell carcinoma, upon further clinical inquiry, we found that the patient had a history of prostate adenocarcinoma 4 years ago, status‐post robotic‐assisted laparoscopic radical prostatectomy. This was followed by adjuvant radiotherapy and hormonal therapy. He started receiving adjuvant androgen deprivation therapy, consisting of leuprolide acetate (Lupron) and bicalutamide (Casodex), 1 year after the operation and continued for a year. The patient was under regular follow‐up care by his uro‐oncology team. As the histopathologic changes were not typical of a classic prostate adenocarcinoma, an immunohistochemical study including PSA and PAP was performed. Both stains, however, yielded negative results (Figure 3B). The core biopsy and subsequent radical prostatectomy specimens were reported as poorly differentiated/undifferentiated carcinoma (Gleason 7–10) with perineural involvement, suspicious vascular/lymphatic invasion, extra‐capsular extension, and positive surgical margins. Bilateral seminal vesicles and bladder were also involved (Gleason3+4, pT3b, R1, pNX, MX). Upon retrospective review, areas with high grade/poor differentiation were detected only focally in the resection specimen with no evidence of squamous differentiation. Given the negative main prostate markers, we evaluated more sensitive prostate immunomarkers, including PSMA and NKX3.1 on the skin biopsy. Cytoplasmic and membranous staining of PSMA, in approximately 50% of the cells with moderate to strong intensity, was present, while 30%–40% of neoplastic cells were moderately to strongly reactive for NKX3.1 in a nuclear pattern. These two immunomarkers confirmed the prostate origin (Figures 3C–F). Although the differential diagnoses for an epidermotropic cancer include malignant melanoma, adenocarcinoma of different origins, pagetoid squamous cell carcinoma, or metastatic squamous cell carcinoma, these entities were ruled out by the positive prostate‐specific immunohistochemical results. Urothelial carcinoma may be another consideration histopathologically; however, PSMA is mostly negative in this tumor [16]. Upon complete skin re‐excision of the involved area on the leg, a residual metastatic lesion in a larger scale was present. There was a rare focus in the dermis, suspicious of perineurial invasion. The same immunoprofile was present in the re‐excision specimen, as expected.

The uro‐oncologic treatment team was notified of this unusual occurrence. Subsequent total body scans and radiographic studies were performed, and no evidence of metastatic disease was found elsewhere. The skin area was then irradiated. On 12 and 24 months interval follow‐ups by his oncologist, the patient was reported to be doing well, with serum PSA levels remaining undetectable at 0.07.

## 3. Discussion

Cutaneous metastasis of prostate adenocarcinoma is quite rare, representing 1% or less of all cutaneous metastases [4]. Cutaneous spread of prostate cancer most often occurs regionally in the inguinal area and the penis (28%), followed by the abdomen (23%), head and neck (16%), chest (14%), extremities (10%), and back (9%) [4]. These cutaneous metastases can occur via direct extension of the malignancy, seeding during surgical resection, or hematogenous/lymphatic spread [17]. It is more common for prostate adenocarcinoma to metastasize to the bone, lymph nodes, lungs, and liver [18]. Cutaneous spread of prostate adenocarcinoma is typically aggressive and has a poor prognosis, as the majority of patients die within 1 year of diagnosis. Metastasis often occurs late in the disease course [19–21]. Clinically, it most frequently manifests as papules and plaques in the inguinal area, bilateral medial thighs, penis, or scrotum [22]. The gross/clinical appearance can be highly variable, and unusual locations may be involved, such as the umbilical area (i.e., Sister Mary Joseph nodule), subungual metastases, clustered, linear/zosteriform, or irregularly distributed papules, and solitary or multiple nodules on the chest, face, and head and neck. [23–31]. The classic histopathology of metastatic prostate adenocarcinoma consists of sheets of undifferentiated cells in the dermis, with nuclear hyperchromasia and prominent eosinophilic nucleoli, and gland‐like structures may form. A Grenz zone may be present, contrary to our findings. The malignant cells may also involve adnexal structures [29]. Squamous differentiation is a very rare phenomenon in prostate cancer. Only rarely in metastatic prostate adenocarcinoma, squamous transformation may occur, especially when hormonal therapies are part of the systemic treatment [15, 32, 33].

Our case is unique from a clinical and histopathological standpoint. A keratotic nodule on the shin is a highly unusual clinical consideration for metastatic prostate cancer, as neither the anatomic location nor the appearance is typical. This lesion could have been easily dismissed by the patient or the physician. Histopathologic features like prominent squamoid appearance, acantholysis, and epidermotropism were certainly not in favor of prostate tissue as the primary source of the metastatic lesion, especially when coupled with negative reactions for PSA or PAP immunostains. Most prostate adenocarcinomas show positivity for both markers. In well‐differentiated prostate cancers, the sensitivity for both markers is almost 100%; however, false‐negative results may occur in 2% to 10% of cases [34]. In poorly differentiated and high‐grade cases of prostate carcinoma, positivity drops to 85% for PSA and to 95% for PAP [35]. Therefore, negative PSA or PAP does not rule out prostate adenocarcinoma. In order to improve the detection of prostate adenocarcinoma, utilization of other sensitive immunomarkers is needed. PSMA, a transmembrane glycoprotein expressed at low levels in benign prostate epithelium, is markedly upregulated in prostate carcinoma. The sensitivity and specificity of PSMA in distinguishing prostate cancer from any other type of malignancy are 65.9% and 94.5%, respectively [36]. The degree of expression of this marker seems to be inversely correlated with the degree of tumor differentiation. NKX3.1, a prostatic tumor suppressor gene located on chromosome 8p, is the newest prostate‐related marker and, unlike other markers, is localized to the nucleus of cells and is highly sensitive for prostate cancer [14, 37, 38]. Most studies have shown that staining for NKX3.1 protein is positive in the majority of primary prostatic adenocarcinomas; however, it may be downregulated in many high‐grade primaries and completely lost in the majority of metastatic prostate carcinomas (e.g., in 65% to 78% of lesions) [14]. In one study, the sensitivity of individual markers in detecting prostate origin in metastatic prostate carcinomas was compared, which showed a detection rate of 99%–100% for NKX3.1, 84.3% for PSMA, 81% for PSA, and 66% for PAP [38]. Our patient’s cutaneous lesion was positive for NKX3.1 (nuclear) and PSMA (membranous and cytoplasmic) in the squamoid areas, despite negative PSA and PAP. Squamous transformation was a separate, unusual, and interesting histomorphologic feature in our case. Squamous cell carcinoma of the prostate is a rare entity. SCC transformed from prostatic adenocarcinoma is even rarer and has been reported following androgen deprivation therapy or radiotherapy in patients with prostate cancer, anywhere from months to years later [15, 32, 33, 39, 40]. As SCC histomorphologically and immunophenotypically is not organ‐specific, confirmation of prostatic origin, especially in evaluating metastatic lesions, requires a strong clinical correlation. We performed a comprehensive search of the medical literature and did not find any cases of primary/denovo squamous cell carcinoma or cutaneous adenosquamous carcinoma reported to express prostate‐specific antigens. Rare cases of metastatic SCC transformed from a prior prostate adenocarcinoma negative for all prostate markers have been reported. After performing comprehensive genomic testing (CGT) and molecular profiling of the metastatic lesion and the original prostatectomy specimen, these reports showed identical somatic mutations and alterations, suggesting a transformation of the patient’s prostate adenocarcinoma into SCC [15, 32, 40, 41]. The proposed mechanism of such a transformation is still unclear, but it has been speculated that the SCC may arise from multipotential prostate adenocarcinoma cells given antecedent hormonal therapy and radiotherapy [15]. The fact that many of the molecular features in the primary adenocarcinoma were retained in the SCC may suggest that transformation may occur through lineage plasticity in response to selective treatment‐related pressure (i.e., the ability of a cell to shift from a committed developmental pathway to another new phenotype) [42]. One limitation of our report is that comparative molecular studies of the patient’s primary and metastatic lesions could not be performed at the time of manuscript preparation.

Other unusual features in our case were the epidermotropism and foci of prominent acantholysis. To our knowledge, there are only two other documented cases of cutaneous metastatic prostate carcinoma with epidermotropism in the published literature [27, 43]. We could not detect a metastatic prostate adenocarcinoma with squamous differentiation and acantholysis in our extended literature search. Acantholysis, primarily a characteristic of squamous cell carcinoma, has been shown as a unique histopathologic feature in nonsquamous cancers such as anaplastic Paget or extramammary Paget disease [44, 45]. While the exact mechanism of acantholysis is unknown, the epidermotropic cancer cells may be able to induce phenotypic/morphologic changes in epidermal cells or somehow damage/impair cell‐to‐cell adhesion, leading to acantholysis. Another hypothesis could be a squamoid change or differentiation in the epidermotropic neoplasm itself, leading to acantholysis. In our case, epidermotropism and acantholysis are features that could have been misinterpreted as primary squamous cell carcinoma. It is imperative that pathologists, dermatopathologists, dermatologists, urologists, and oncologists are aware of such rare and unusual cases of metastatic prostatic lesions. Providing a thorough clinical history, including prior cancer diagnoses and treatment modalities is of paramount importance. When feasible, comparative genomic profiling of the primary and metastatic lesions, is highly valuable for confirming the origin of a metastatic skin lesion.

Although genomic classifiers/biomarkers have shown promise in risk stratification, no validated biologic marker currently predicts the metastatic potential for prostate cancer. Current surveillance primarily relies on serial serum PSA measurements, following surgical interventions and definitive treatments. In light of current knowledge, thorough clinical skin examinations, with careful evaluation of any newly developing cutaneous lesions, should be offered and incorporated into the regular clinical follow‐up of these patients [46].

## Funding

No funding was received for this manuscript.

## Consent

The patient consent form is kept on file, although no patient identifier was used in this paper.

## Conflicts of Interest

The authors declare no conflicts of interest.

## Data Availability

The data that support the findings of this study are available from the corresponding author upon reasonable request.
